# Antioxidant, Anti-α-Glucosidase, Anti-Tyrosinase, and Anti-Acetylcholinesterase Components from Stem of *Rhamnus formosana* with Molecular Docking Study

**DOI:** 10.3390/antiox14010008

**Published:** 2024-12-24

**Authors:** Chia-Hsuan Tsai, Ya-Lun Liou, Sin-Min Li, Hsiang-Ruei Liao, Jih-Jung Chen

**Affiliations:** 1 Department of Plastic and Reconstructive Surgery, Keelung Chang Gung Memorial Hospital, Keelung 204201, Taiwan; chtsai0715@gmail.com; 2 College of Medicine, Chang Gung University, Taoyuan 333323, Taiwan; 3 Department of Pharmacy, School of Pharmaceutical Sciences, National Yang Ming Chiao Tung University, Taipei 112304, Taiwan, China; muset311386@gmail.com (Y.-L.L.); samuel147samuel147@gmail.com (S.-M.L.); 4 Graduate Institute of Natural Products, College of Medicine, Chang Gung University, Taoyuan 333323, Taiwan, China; 5 Graduate Institute of Biomedical Sciences, College of Medicine, Chang Gung University, Taoyuan 333323, Taiwan, China; 6 Department of Anesthesiology, Chang Gung Memorial Hospital, Taoyuan 333323, Taiwan; 7 Department of Medical Research, China Medical University Hospital, China Medical University, Taichung 404333, Taiwan; 8 Traditional Herbal Medicine Research Center, Taipei Medical University Hospital, Taipei 110301, Taiwan

**Keywords:** *Rhamnus formosana*, antioxidant, anti-α-glucosidase, anti-acetylcholinesterase, anti-tyrosinase, molecular docking

## Abstract

*Rhamnus formosana* is a creeping evergreen shrub endemic to Taiwan. In traditional medicine, Rhamnaceae plants are used as herbal remedies for conditions such as itching, difficulty urinating, and constipation. This study explores the inhibitory effects of various solvent extracts and bioactive components of *R. formosana* on α-glucosidase, tyrosinase, acetylcholinesterase (AChE), and antioxidant activity. The 100 °C water extract exhibited strong antioxidant activity in DPPH, ABTS, superoxide, and FRAP assays. The methanol extract demonstrated the highest α-glucosidase inhibitory effect, while the ethanol extract displayed potent AChE inhibition and the acetone extract showed the most potential tyrosinase inhibitory activity among the extracts. Five main biocomponents were isolated and evaluated for their bioactivities. Among them, kaempferol (**1**) and quercetin (**2**) exhibited notable antioxidant activity in DPPH and ABTS assays. Particularly, kaempferol (**1**) performed the best *α*-glucosidase inhibitory effect, physcion (**5**) showed the strongest AChE inhibition, and quercetin (**2**) demonstrated the most potential for tyrosinase inhibitory activity. Further molecular docking studies revealed that there may be stronger binding mechanisms between bioactive components and target enzymes (including α-glucosidase, acetylcholinesterase, and tyrosinase) than the positive control. These findings suggest that bioactive extracts and compounds from the stems of *R. formosana* may have potential as natural antioxidant, anti-α-glucosidase, anti-AChE, and anti-tyrosinase drug candidates or dietary supplements for the management of oxidative stress-related conditions, including hyperglycemia, pigmentation disorders, and neurodegenerative diseases.

## 1. Introduction

Rhamnaceae plants are widely used in traditional Asian medicine and are known for their therapeutic applications against itching, urinary disorders, and digestive issues. Many plants in this family are rich in bioactive compounds, including polyphenols, flavonoids, anthraquinones, and naphthols, which have various physiological functions such as antioxidant, anticancer, hypoglycemic, and anti-acetylcholinesterase activities [1]. In particular, flavonoids are recognized as the primary antioxidant components in plants, helping to alleviate oxidative stress caused by free radicals. Anthraquinones are primarily known for their laxative, anticancer, and antimicrobial activities. Numerous studies have been conducted on different species of the family Rhamnaceae around the world, demonstrating their potential as moderate acetylcholinesterase (AChE) inhibitors through interactions facilitated by their aromatic structures [2].

In the body, an imbalance between reactive oxygen species (ROS) production and antioxidant defense mechanisms results in the oxidative stress, leading to damage involved in the pathogenesis of various chronic diseases, such as cardiovascular disease, diabetes, neurodegenerative diseases, and cancer. Antioxidants play a crucial role in neutralizing ROS, thereby preventing cell damage and maintaining physiological homeostasis [3]. Given the rising interest in natural antioxidants as safer alternatives to synthetic ones, plants with bioactive compounds, especially polyphenols and flavonoids, are being actively explored.

Diabetes mellitus (DM) is a chronic metabolic disease characterized by elevated blood glucose levels (hyperglycemia) resulting from defects in insulin secretion, insulin action, or both in the metabolism of carbohydrates, fats, and proteins. It is estimated that by 2030, more than 366 million people worldwide will be affected by diabetes, making it one of the most concerning health issues globally and leading to microvascular diseases (such as retinopathy, neuropathy, and kidney disease) and macrovascular diseases (including heart disease, stroke, and peripheral vascular disease). Diabetes management is conducted through treatment with insulin, oral hypoglycemic drugs, regular physical activity, healthy diet, and psychosocial care. However, while many existing antidiabetic drugs effectively control blood glucose levels, they are associated with adverse side effects [4].

Alzheimer’s disease (AD) is a neurodegenerative disorder that primarily affects the brain and is the most common cause of dementia. It leads to the deterioration of critical cognitive functions such as memory, comprehension, and speech. Additionally, symptoms may include behavioral changes such as anxiety and restlessness. The destruction of cholinergic neurons leads to the depletion of the neurotransmitter acetylcholine (ACh), which plays a crucial role in cognitive processes such as learning and memory. Therefore, the most promising approach to symptomatic treatment and slowing the progression of AD is the use of cholinesterase inhibitors, which work by inhibiting acetylcholinesterase (AChE) and increasing the levels of the neurotransmitter in the brain. However, the use of cholinesterase inhibitors can cause side effects, mainly gastrointestinal problems such as nausea, vomiting, and diarrhea [5].

The skin is an important barrier that protects the body from harmful environmental toxins as well as physical, biological, and environmental attacks. While sun exposure can provide health benefits, excessive ultraviolet (UV) radiation, particularly UV-A, can disrupt epidermal cell metabolism, leading to photoaging, carcinogenesis, erythema, acute pigment darkening, and persistent pigment darkening. UV-A exposure induces the production of reactive oxygen species (ROS), such as singlet oxygen and hydroxyl radicals, which damage proteins, lipids, and DNA, and trigger inflammatory responses. Furthermore, UV-A can increase the activity of tyrosinase, a key enzyme in melanin production, leading to excessive pigmentation and potential skin lesions. To mitigate these effects, flavonoids and phenolic acids from plant extracts are widely used in cosmetics to inhibit tyrosinase activity and reduce UV absorbing [6].

Today, natural products encompassing herbs, herbal mixtures, dietary supplements, Traditional Chinese Medicine (TCM), and other alternative therapies are widely recognized for their medicinal potential. In our past research, many potent bioactive components from natural sources have been discovered [7,8,9,10,11,12]. Despite having identified various components of flavonoids and anthraquinones, *Rhamnus formosana* has not been extensively studied for its application and bioactivity. Given the global rise in oxidative stress-related disorders such as diabetes, pigmentation issues, and neurodegenerative diseases, as well as the potential bioactive properties of *Rhamnus formosana*, this study aims to evaluate the antioxidative and enzyme inhibitory properties of *Rhamnus formosana* extracts and isolated compounds, providing insights into its potential as a functional ingredient for health and wellness applications. Furthermore, molecular docking is conducted to investigate the binding mechanism between potential components and enzymes, including α-glucosidase, acetylcholinesterase, and tyrosinase.

## 2. Materials and Methods

### 2.1. Experimental Chemicals

Tyrosinase, AChE ABTS, bovine serum albumin, TPTZ, trolox, α-glucosidase, Folin-Ciocalteu’s reagent, chlorogenic acid, acetylcholine iodide and DTNB were obtained from commercial suppliers (Sigma-Aldrich, St. Louis, MO, USA). We procured Arbutin and quercetin by AK scientific and MedChemExpress (Monmouth Junction, NJ, USA), respectively. Potassium peroxodisulfate, disodium hydrogen phosphate, sodium dihydrogen phosphate, and sodium carbonate were purchased from SHOWA Chemical Co. Ltd. (Chuo-ku, Japan). We obtained ferric chloride (FeCl_3_), aluminum chloride (AlCl_3_), and (*p*-NPG) *p*-nitro-phenyl-α-D-glucopyranoside from Alfa Aesar (Lancashire, UK). Phenazine methosulphate (PMS), nitroblue tetrazolium (NBT), and DPPH were supplied by Tokyo Chemical Industry Co., Ltd. (Tokyo, Japan). Acarbose, sodium acetate, potassium acetate, butylated hydroxytoluene (BHT), and nicotinamide adenine dinucleotide (NADH) were obtained from (Acros Organics, Giel, Belgium). Acetic acid was sourced from Avantor Performance Materials, LLC, Radnor, PA, USA.

### 2.2. Crude Extract of Rhamnus formosana

We purchased *Rhamnus formosana* stems from Wanhua District, Taipei City, Taiwan in February 2024, and they were distinguished by Professor J.-J. Chen. Samples were deposited at the Department of Pharmacy, National Yang Ming Chiao Tung University, Taipei, Taiwan. *Rhamnus formosana* stems (57.0 g) were immersed in 250 mL of various solvents (*n*-hexane, acetone, ethyl acetate, methanol, ethanol, water, and 100 °C water), and then shaken at room temperature for 1 h. Various extracts were filtered through Whatman No. 1 (filter paper) before being concentrated using the rotary evaporator at 38 °C to obtain the dried solvent extracts. The yields of the extracts were as follows: *n*-hexane (0.23%), acetone (1.54%), ethyl acetate (2.79%), methanol (2.91%), ethanol (7.51%), water (4.09%), and 100 °C water (9.85%). The extracts were stored at −20 °C until further analyses.

### 2.3. Preparation of Active Components of Rhamnus formosana

**Figure 1:**
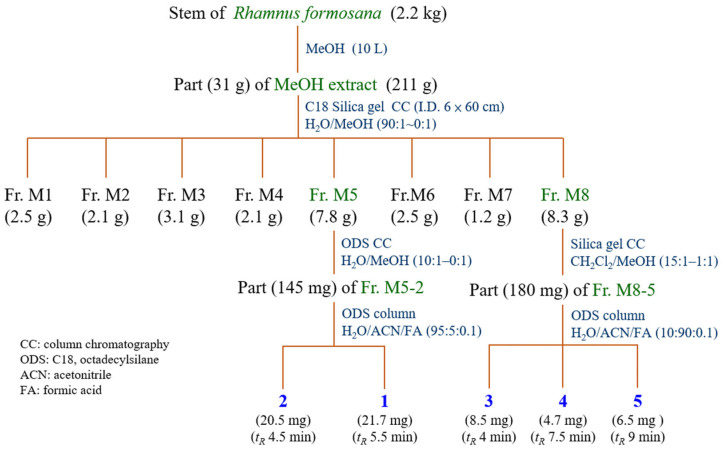
Extraction and isolation of active ingredients from *Rhamnus formosana*.

**Figure 2:**
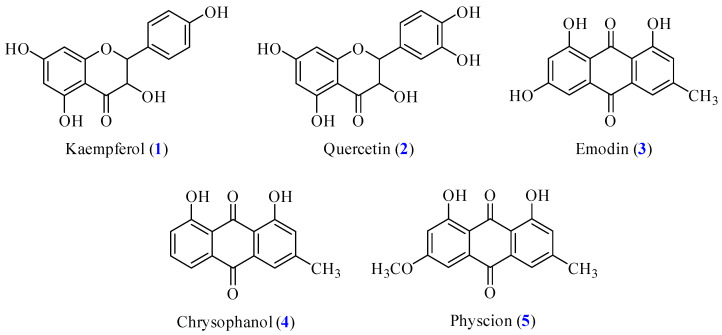
Chemical structures of kaempferol (**1**), quercetin (**2**), emodin (**3**), chrysophanol (**4**), and physcion (**5**) from *Rhamnus formosana*.

The plant material was shade-dried, crushed, and stored in airtight containers for further use. A mobile phase consisting of 0.1% formic acid aqueous solution (*v*/*v*) (solvent A) and acetonitrile (solvent B) was used for reversed-phase HPLC separation [13]. The powdered *Rhamnus formosana* (2.2 kg) was soaked in 100% methanol (10 L) for 3–4 days with intermittent shaking for 3 h (Figure 1). The extract was filtered and concentrated under reduced pressure at 37 °C to provide a methanol-soluble fraction (Fraction M; 211 g). Part (31 g) of fraction M was purified by C18 silica gel column chromatography (I.D. 6 × 50 cm; H_2_O/MeOH gradient) to afford 8 fractions: Fr. M1–M8. Fraction M5 (7.8 g) was subjected to ODS column chromatography (H_2_O/MeOH 10:1–0:1) to result in 9 subfractions: M5-1–M5-9. Part (145 mg) of M5-2 was purified by ODS column (95% A and 5% B for 40 min) to obtain Compounds **1** (21.7 mg, t_R_ = 5.5 min) and **2** (20.5 mg, t_R_ = 4.5 min). Fraction M8 (8.3 g) was subjected to silica gel column chromatography (CH_2_Cl_2_/MeOH 15:1–1:1) to produce 8 subfractions: M8-1–M8-8. Part (180 mg) of M5-2 was purified by ODS column (10% A and 90% B for 40 min) to obtain Compounds **3** (8.5 mg, t_R_ = 4 min), **4** (4.7 mg, t_R_ = 7.5 min), and **5** (6.5 mg, t_R_ = 9 min). The isolated compounds, including kaempferol (**1**), quercetin (**2**), emodin (**3**), chrysophanol (**4**), and physcion (**5**), were identified by NMR (Figures S1–S5), and the structures are shown in Figure 2.

### 2.4. Quantification of Total Phenolic Content

The experimental procedure for TPC analysis of each solvent extract was determined based on the previous reported method [14]. The extract was diluted to 100 μg/mL with deionized water. Then, 200 μL of the tested sample was combined with 200 μL of Folin–Ciocalteu reagent (0.5 N, previously diluted with deionized water) in sealed tubes. Following this, 400 μL of 20% Na_2_CO_3_ solution was added and incubated in the dark for 40 min, and the absorbance was measured at 750 nm. The TPC of the extracts was calculated and the results were expressed as milligrams of gallic acid equivalent (GAE) per gram of extract. A standard curve was generated using gallic acid.

### 2.5. Quantification of Total Flavonoid Content

The TFC of each solvent extract was determined according to previously reported procedures [15]. The extracts were diluted to 100 μg/mL with MeOH. In each sealed tube, 200 μL of the diluted extract and quercetin solution were combined with 100 μL of a 10% AlCl_3_ solution and 100 μL of 0.1 mM potassium acetate. The samples were then incubated for 30 min, and the absorbance was recorded at 415 nm. TFC was calculated and reported as milligrams of quercetin equivalents (QEs) per gram of extract. A quercetin standard curve was established for quantification.

### 2.6. Scavenging Activity of DPPH Free Radical

DPPH analysis was performed according to the previously published procedure [16]. A 400 μM DPPH radical solution was prepared in EtOH. The tested samples were dissolved in DMSO to prepare stock solutions, which were then diluted to the desired concentrations with EtOH. In a 96-well plate, 100 μL of each diluted tested sample solution was mixed with 100 μL of the DPPH radical solution (200 μM). The mixture was allowed to react in the dark at room temperature for 30 min. After the reaction, the absorbance at 520 nm was measured using an ELISA reader. DPPH radical scavenging activity was calculated by the following equation:

(1) $$\mathrm{Scavenging}\;\mathrm{rate}\;(\%)=\frac{{(OD}_{0}-{OD}_{1})}{{OD}_{0}}$$

where OD_0_ is absorbance of the control and OD _1_ is that of the tested sample.

### 2.7. Scavenging Activity of ABTS Radical

The analysis of ABTS was performed according to the previously published method [17]. The ABTS solution (28 mM) was mixed with potassium permanganate solution (9.6 mM) in a 1:1 ratio (*v*/*v*) and allowed to react in the dark at room temperature for 16 h to generate the ABTS radical solution. The ABTS radical solution was then diluted with EtOH until the absorbance at 740 nm reached 0.70 ± 0.02. The tested samples were dissolved in DMSO to prepare stock solutions, which were subsequently diluted to the desired concentrations with EtOH. In a 96-well plate, 190 μL of the diluted ABTS radical solution was mixed with 10 μL of each diluted sample solution. The mixture was allowed to react in the dark at room temperature for 6 min. The absorbance at 740 nm was then measured using an ELISA reader. The ABTS radical scavenging activity was calculated by the following equation:

(2) $$\mathrm{Scavenging}\;\mathrm{rate}\;(\%)=\frac{{(OD}_{0}-{OD}_{1})}{{OD}_{0}}\times 100$$

where OD_0_ is absorbance of the control and OD_1_ is that of the tested sample.

### 2.8. Scavenging Activity of Superoxide Radical

The superoxide scavenging activity assay was performed based on the published method [16]. The superoxide radical was prepared in a 16 mM Tris-HCl buffer (pH 8.0) containing 50 µL of NBT (300 µM), 50 µL of PMS (120 µM), and 50 µL of different concentrations of the tested sample. The reaction was initiated by adding 50 µL of the NADH (468 µM) solution to the mixture and incubated at room temperature for 5 min. The activity of the samples was determined by calculating the decrease in absorbance measured at 560 nm by the following equation:

(3) $$\mathrm{Scavenging}\;\mathrm{rate}\;(\%)=\frac{{(OD}_{0}-{OD}_{1})}{{OD}_{0}}\times 100$$

where OD_0_ is absorbance of the control and OD_1_ is that of the tested sample.

### 2.9. Ferric Reducing Antioxidant Power (FRAP)

The analysis was performed using the published method [18]. The working solution was prepared by mixing acetate buffer (pH 3.6), ferric chloride solution (20 mM), and TPTZ solution (10 mM TPTZ in 40 mM HCl) in a 10:1:1 ratio. Then, 900 µL of this solution was warmed to 37 °C and mixed with 100 µL of the diluted sample, blank, or standard in a microcentrifuge tube. The tubes were vortexed and incubated in a dry bath at 37 °C for 40 min. Absorbance was measured at 593 nm. The standard curve was linear from 0 to 100 mM Trolox, and results were expressed as mM TE/g dry weight.

### 2.10. α-Glucosidase Inhibitory Activity Assessment

α-Glucosidase inhibition was determined by previously reported methods [19]. The α-glucosidase solution was diluted with 0.1 M sodium phosphate buffer (pH 6.8) to 1 U/mL. Then, the tested sample was mixed with 20 µL of α-glucosidase solution in a microcentrifuge tube. Following that, 380 µL of the substrate *p*-NPG (0.53 mM) was added, and the mixture was vortexed and incubated at 37 °C for 40 min in a dry bath. The reaction was stopped by adding 500 µL of 0.1 M Na_2_CO_3_ solution. The absorbance of the released *p*-nitrophenol (*p*-PNP) was measured at 400 nm.

### 2.11. Tyrosinase Inhibitory Activity Analysis

The method for determining tyrosinase inhibition was carried out according to the reported literature [20]. The substrate was premixed with the sample at various concentrations in potassium phosphate buffer (pH 6.5, 50 mM) and incubated at room temperature for 10 min. Then, 20 µL of mushroom tyrosinase (1000 units/mL) was added, and the mixtures were incubated for an additional 30 min. Absorbance was measured at 490 nm, and the percentage of tyrosinase inhibition was calculated by the following equation:

(4) $$\mathrm{Inhibition}\;(\%)=\frac{{(OD}_{0}-{OD}_{1})}{{OD}_{0}}\times 100$$

where OD_0_ is absorbance of the control and OD_1_ is that of the tested sample.

### 2.12. Acetylcholinesterase Inhibitory Activity Analysis

Determination of AChE inhibition was conducted based on the reported method with slight modifications [21]. Sodium phosphate buffer (0.1 M, pH 8.0, 140 µL), test compound solution (20 µL), enzyme solution (15 µL of AChE at 0.2 units/mL), and DTNB (15 mM, 10 µL) were mixed and incubated for 10 min at room temperature. The reaction was then initiated by adding 10 µL of the substrate (AChI, 15 mM). Finally, the reaction mixture was incubated at 20 °C for 10 min, and the absorbance was measured at 405 nm.

### 2.13. Molecular Docking Studies

Molecular docking analysis of the compounds was performed using a previously reported procedure [21]. The binding affinity of the bioactive compounds and target enzymes was calculated using Biovia Discovery Studio Client 2021 software. The molecular structures were downloaded from the PubChem SDF database and pasted into ChemDraw Ultra 16.0. The minimum energy was then calculated using Chem3D, and the structure was saved in mol2 format. Ligands were prepared using the Autodock suite by adding hydrogen and charges (Gasteiger). Discovery Studio was used to view the molecule size ranges, after which hydrogen atoms were removed from the protein molecules. The processed compound structures were imported into Autodock Vina for molecular docking analysis. Multiple docking positions were calculated and ranked based on binding energy. Optimal docking poses were visualized and analyzed to identify key binding amino acid residues [22].

### 2.14. Statistical Analysis System

All experimental data are presented as mean ± SEM. Shapiro–Wilk test was performed using IBM SPSS Statistics (version 29.0) before Student’s *t*-test. Statistical analysis of Student’s *t*-test was performed with a *p*-value of 0.05 or less considered statistically significant. All analyses were conducted at least three times.

## 3. Results

### 3.1. Quantification of TPC, TFC, and Extraction Yields per Solvent of Rhamnus formosana

The stems of *R. formosana* were extracted using various solvents, including 100 °C water, water, methanol, ethanol, acetone, ethyl acetate, and *n*-hexane. The TPC, TFC, and extract yields of each extract were determined and are shown in Table 1. The extraction yields ranged from 0.23% (*n*-hexane) to 9.85% (100 °C water), and the yield of 100 °C water was significantly higher than other solvents. Additionally, the results indicated a trend where the yield decreased as solvent polarity decreased. Among the solvents, the acetone extract exhibited the highest TPC (64.42 ± 0.41 mg/g), followed by ethanol (59.98 ± 3.87 mg/g) and ethyl acetate (58.64 ± 4.39 mg/g). Interestingly, the TFC results showed a slightly different pattern. The EtOAc extract (25.07 ± 0.99 mg/g) was found to possess the highest TFC, followed by *n*-hexane (20.67 ± 3.13 mg/g) and acetone (17.60 ± 3.12 mg/g).

### 3.2. Scavenging Activity of DPPH

Table 2 presents the DPPH free radical scavenging activity of each extract of *R*. *formosana*. Compared to the positive control BHT (SC_50_ = 156.04 ± 14.31 μg/mL), all solvent extracts demonstrated superior scavenging activity than the BHT. The 100 °C water extract exhibited the strongest DPPH free radical inhibition activity (SC_50_ = 21.34 ± 0.52 μg/mL), followed by the water extract (SC_50_ = 26.03 ± 1.95 μg/mL) and the ethyl acetate (EtOAc) extract (SC_50_ = 34.91 ± 6.43 μg/mL).

### 3.3. Scavenging Activity of ABTS

As shown in Table 2, although the ABTS radical scavenging activities of the different solvent extracts differed slightly, their performance was comparable to the positive control (BHT, SC_50_ = 9.12 ± 1.85 μg/mL). The 100 °C water extract demonstrated relatively strong ABTS scavenging activity (SC_50_ = 13.63 ± 2.31 μg/mL), followed by the EtOAc extract (SC_50_ = 13.94 ± 3.26 μg/mL), acetone extract (SC_50_ = 14.51 ± 1.19 μg/mL), and ethanol extract (SC_50_ = 14.52 ± 0.83 μg/mL). The methanol extract (SC_50_ = 18.35 ± 0.67 μg/mL) also exhibited potential free radical scavenging effects.

### 3.4. Scavenging Activity of Superoxide Radical

As shown in Table 2, the positive control, cynaroside, showed a SC_50_ value of 12.03 ± 4.20 μg/mL in the superoxide radical scavenging test. In comparison, the water extract (SC_50_ = 28.92 ± 2.37 μg/mL) exhibited the most significant effect among the extracts tested, followed by the 100 °C water extract (SC_50_ = 41.84 ± 1.40 μg/mL) and the acetone extract (SC_50_ = 50.26 ± 6.85 μg/mL). However, the *n*-hexane (SC_50_ > 400 μg/mL) extract was unfavorable in superoxide radical scavenging.

### 3.5. Ferric Reducing Antioxidant Power

As shown in Table 2, the ethanol extract exhibited the highest ferric reducing antioxidant power (TE = 1463.77 ± 25.30 mM/g), followed by the 100 °C water extract (TE = 1307.21 ± 4.19 mM/g), methanol extract (TE = 1273.00 ± 26.26 mM/g), acetone extract (TE = 1252.35 ± 121.86 mM/g), and water extract (TE = 1229.73 ± 80.01 mM/g). Conversely, the *n*-hexane extract (TE = 19.82 ± 4.91 mM/g) and the EtOAc extract (TE = 1096.56 ± 100.09 mM/g) demonstrated relatively lower reducing abilities.

Based on the antioxidation results including DPPH, superoxide radical scavenging activity, and FRAP analysis, five of the seven *R. formosana* extracts exhibited strong antioxidant capacity. These differences in inhibition abilities could be attributed to the varying TPC or TFC in each solvent extract. In the DPPH and ABTS assays, the 100 °C water extract showed the strongest performance, while the water extract was most effective in the superoxide radical scavenging assay. In the FRAP test, ethanol extract exhibited the highest antioxidant activity.

### 3.6. Determination of Inhibitory Activity Against α-Glucosidase

The results in Table 3 show that the methanol extract (IC_50_ = 2.51 ± 0.81 μg/mL) of *R. formosana* demonstrated the strongest α-glucosidase inhibitory activity, followed by the ethyl acetate (EtOAc) extract (IC_50_ = 3.19 ± 0.40 μg/mL), ethanol extract (IC_50_ = 3.88 ± 1.06 μg/mL), acetone extract (IC_50_ = 4.13 ± 0.53 μg/mL), 100 °C water extract (IC_50_ = 7.88 ± 0.38 μg/mL), and water extract (IC_50_ = 8.04 ± 1.72 μg/mL). Meanwhile, the *n*-hexane extract showed lower inhibition (IC_50_ = 36.35 ± 6.00 μg/mL). Interestingly, all solvent extracts exhibited significantly better α-glucosidase inhibition than the antidiabetic drug acarbose (IC_50_ = 523.62 ± 76.25 μg/mL).

### 3.7. Determination of the Inhibitory Activity Against Acetylcholinesterase (AChE)

All solvent extracts were also tested for their AChE inhibitory effects, and the results are shown in Table 3. The ethanol extract demonstrated the strongest AChE inhibitory activity (IC_50_ = 63.23 ± 3.97 μg/mL) among the solvent extracts, followed by acetone (IC_50_ = 84.66 ± 4.18 μg/mL), ethyl acetate (IC_50_ = 90.36 ± 7.28 μg/mL), and water (IC_50_ = 169.66 ± 8.85 μg/mL). Importantly, most of these extracts showed stronger inhibition than the positive control, chlorogenic acid (IC_50_ = 175.89 ± 12.33 μg/mL). However, the 100 °C water extract (IC_50_ = 190.17 ± 11.26 μg/mL) was unfavorable to AChE inhibition and demonstrated lower inhibition than chlorogenic acid.

### 3.8. Determination of Inhibitory Activity Against Tyrosinase

Among all the extracts, the results in Table 4 indicate that the ethyl acetate extract demonstrated the highest anti-tyrosinase activity (IC_50_ = 310.16 ± 10.79 μg/mL). However, its activity was still lower than that of the positive control (arbutin, IC_50_ = 162.42 ± 2.77 μg/mL). Additionally, the IC_50_ values of *n*-hexane, methanol, 100 °C water, and water extracts were also higher than 400 μg/mL. Consequently, no further experiments were conducted with these extracts.

Based on the above study, we provided a comparative evaluation of the α-glucosidase, AChE, and tyrosinase inhibitory activities of *R*. *formosana* extracts in different solvents. According to the results, the methanol extract showed the highest potential for inhibiting α-glucosidase, the ethanol extract exhibited the strongest inhibition against AChE, and acetone showed the most potential in tyrosinase inhibition.

### 3.9. Antioxidative Inhibitory Activities of Isolated Components

For further investigating the potential applications of the isolated main compounds (Figure 2), kaempferol (**1**), quercetin (**2**), emodin (**3**), chrysophanol (**4**), and physcion (**5**) were isolated and evaluated for their bioactivities. First, the ABTS and DPPH radical scavenging activities, superoxide radical scavenging, and FRAP were analyzed. The results are presented in Table 5. In the DPPH assay, quercetin (**2**) (SC_50_ = 16.05 ± 2.62 μM) and kaempferol (**1**) (SC_50_ = 61.74 ± 7.36 μM) showed significantly higher radical scavenging activities than the positive control BHT (SC_50_ = 708.15 ± 10.82 μM). A similar trend was observed in the ABTS assay, where quercetin (**2**) (SC_50_ = 5.70 ± 0.92 μM) demonstrated notable radical scavenging activity, followed by kaempferol (**1**) (SC_50_ = 15.32 ± 0.94 μM) and better than the BHT (SC_50_ = 37.50 ± 4.31 μM). In addition, quercetin (**2**) (SC_50_ = 85.80 ± 4.76 μM) also showed the most effective antioxidant activity in the superoxide radical scavenging analysis. The antioxidant activities of the isolated bioactive compounds were further assessed by FRAP analysis. Compared with BHT (TE = 5712.61 ± 176.27 mM/g), kaempferol (**1**) (TE = 1046.16 ± 23.78 mM/g) and quercetin (**2**) (TE = 1026.73 ± 16.69 mM/g) demonstrated moderate antioxidant power. These results indicate that the flavonoids are more favorable for antioxidant activity than anthraquinones.

### 3.10. α-Glucosidase Inhibitory Activity of Isolated Fractions

Five main Compounds **1**–**5** were isolated from *R. formosana* and subjected to evaluate their inhibitory effects on α-glucosidase activity (Table 6). According to the results, kaempferol (**1**) (IC_50_ = 45.54 ± 3.20 μM) showed the strongest α-glucosidase inhibitory activity, followed by emodin (**3**) (IC_50_ = 58.85 ± 3.39 μM) and quercetin (**2**) (IC_50_ = 78.03 ± 8.69 μM), significantly supervising the positive control acarbose (IC_50_ = 406.91 ± 14.48 μM). In contrast, chrysophanol (**4**) (IC_50_ = 604.33 ± 44.82 μM) and physcion (**5**) (IC_50_ > 800 μM) exhibited unfavorable inhibition against *α*-glucosidase.

### 3.11. Acetylcholinesterase (AChE) Inhibitory Activity of Isolated Compounds

Further, Compounds **1**–**5** were subjected to AChE inhibition analysis, and chlorogenic acid was used as a positive control. As shown in Table 6, physcion (**5**) demonstrated the strongest inhibitory activity (IC_50_ = 75.97 ± 1.34 μg/mL), followed by emodin (**3**) (IC_50_ = 77.56 ± 6.28 μg/mL), quercetin (**2**) (IC_50_ = 78.11 ± 2.87 μg/mL), and kaempferol (**1**) (IC_50_ = 86.86 ± 4.62 μg/mL), supervising the positive control, chlorogenic acid (IC_50_ = 302.83 ± 25.24 μM). However, chrysophanol (**4**) (IC_50_ > 400 μM) exhibited unfavorable inhibition against AChE.

### 3.12. Tyrosinase Inhibitory Activities of Isolated Components

The tyrosinase inhibitory activity of isolated Compounds **1**–**5** from *Rhamnus formosana* are shown in Table 7. The results indicated that quercetin (**2**) (IC_50_ = 12.71 ± 1.96 μM) and kaempferol (**1**) (IC_50_ = 19.45 ± 1.82 μM) possessed approximately 33 to 50 times stronger inhibitory activity than the positive control, arbutin (IC_50_ = 638.73 ± 54.96 μM), while anthraquinones **3**–**5** were unfavorable for tyrosinase inhibition.

These results indicate that flavonoids such as kaempferol (**1**) and quercetin (**2**) were more suitable for inhibitions of α-glucosidase and tyrosinase, while anthraquinones such as emodin (**3**) and physcion (**5**) were more favorable in AChE inhibition.

### 3.13. Molecular Modeling Docking

To further investigate the effects of the active compounds on α-glucosidase active binding site, kaempferol (**1**), quercetin (**2**), emodin (**3**), and chrysophanol (**4**) were subjected to molecular docking study by the Discovery Studio 2021 (Accelrys, San Diego, CA, USA) modeling program. Since the 3D crystal structure of *Saccharomyces cerevisiae* α-glucosidase is unavailable [23], the crystal structure of *S. cerevisiae* is used (PDB ID: 3A4A), as it shares 72% sequence homology with α-glucosidase and is commonly utilized in binding studies [24]. This study uses the 3D crystal structure (PDB ID: 3A4A) as a docking model, given that its active site configuration closely resembles that of β-glucosidase in *B. vulgaris*, though it is deeper and narrower [25].

Based on molecular docking results (Table 8), the docking scores of kaempferol (**1**) (−8.6 kcal/mol) and emodin (**3**) (−7.7 kcal/mol) were significantly higher than those of the other active components and acarbose (−4.8 kcal/mol). The strong binding capacity between ligand and active binding site indicated the effective α-glucosidase inhibition, consistent with the inhibitory data (Table 6). Additionally, the docking scores aligned closely with the findings in Table 8. These results suggest that active kaempferol (**1**) and emodin (**3**) may be promising candidates for further investigation as natural α-glucosidase inhibitors.

**Figure 3:**
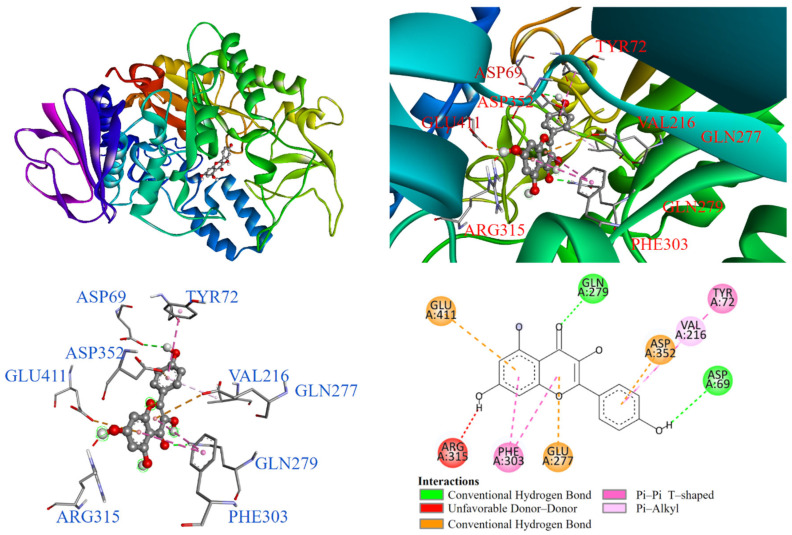
Interactions of kaempferol (**1**) with α-glucosidase active binding site.

**Figure 4:**
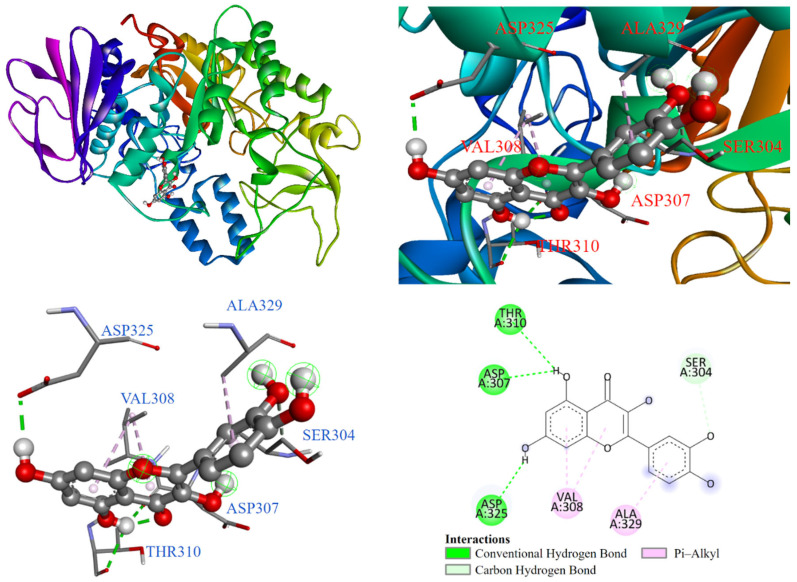
Interaction of quercetin (**2**) with α-glucosidase active binding site.

**Figure 5:**
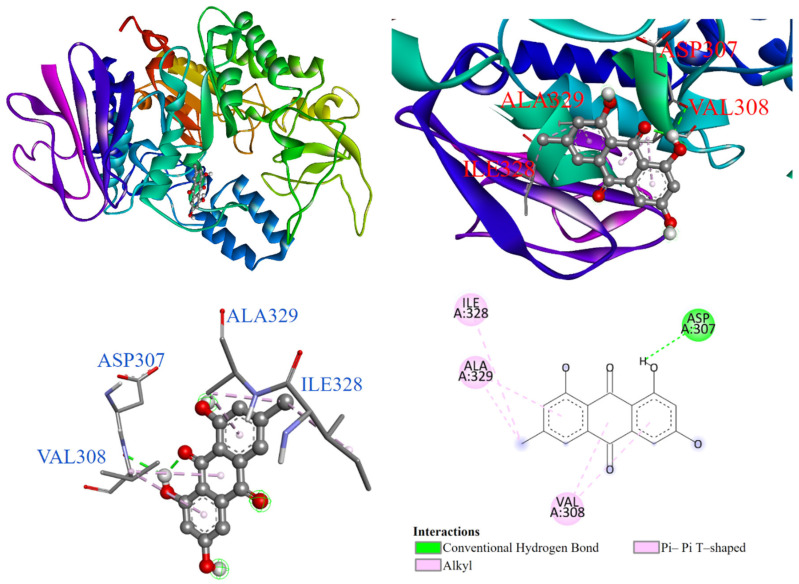
Interaction of emodin (**3**) with α-glucosidase active binding site.

**Figure 6:**
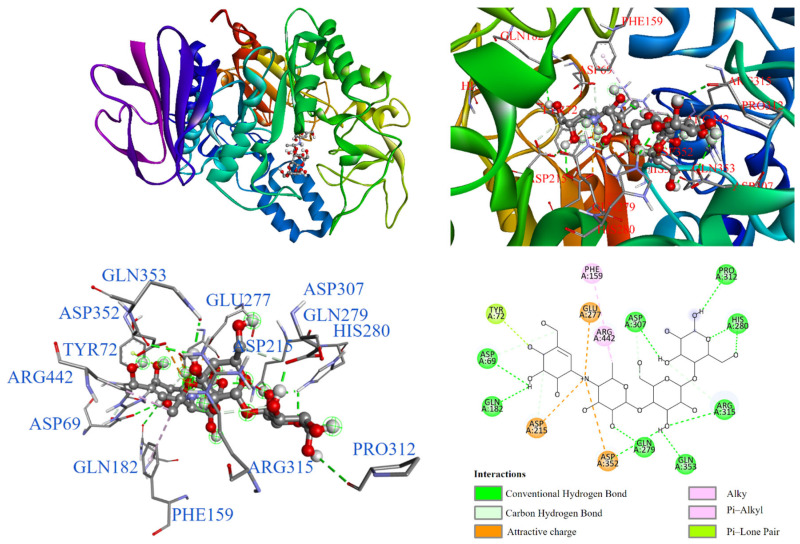
Interaction of acarbose with α-glucosidase active binding site.

Further, molecular docking results for potential Compounds **1**–**3** are visualized in Figure 3, Figure 4 and Figure 5. Molecular docking of acarbose was also conducted, and it is shown in Figure 6 for comparison. As shown in Figure 6, acarbose occupied the deep pocket of the α-glucosidase active site at lower and middle positions. The interaction between acarbose and α-glucosidase suggested that the hydroxyl group in the A-ring pocket of acarbose acts as a hydrogen bond donor, interacting with TYR72 through a π-lone pair interaction. The amine group connecting the A and B rings formed attractive charge interactions with GLU277, ASP215, and ASP352. Additionally, the hydroxyl groups on rings A, B, C, and D formed conventional hydrogen bonds with ASP69, GLN182, GLN279, GLN353, ARG315, HIS280, PRO312, and ASP307. Carbon-1 of acarbose also interacted with alkyl and π-alkyl groups with PHE159 and ARG442.

The structures of polysaccharides and acarbose showed notable similarities, with one key difference being the linkage between the A and B rings. The mechanism of α-glucosidase suggested that the conversion of polysaccharides into monosaccharides involved the cleavage of α-1,4-glycosidic bonds. In acarbose, this linkage involved nitrogen instead of oxygen, resulting in a stronger hydrogen bonding effect due to nitrogen’s presence. Consequently, acarbose remains in the active site for an extended duration, enhancing its ability to inhibit α-glucosidase [26].

On the other hand, Figure 3 illustrates that kaempferol (**1**) entered the α-glucosidase active site. The benzene ring interacted with TYR72 through a π-π T-shaped interaction and with VAL216 via a π-alkyl interaction. The hydroxyl group on the benzene ring formed a conventional hydrogen bond with ASP69. Additionally, the benzene ring of the chromone structure interacted with PHE303 in a π-π T-shaped interaction. The hydroxyl group on the benzene ring acted as a hydrogen bond donor, while it also formed an unfavorable donor–donor interaction with ARG315. The pyrone rings of the chromone structure interacted with GLU277 and GLN279.

As shown in Figure 4, the molecular docking model demonstrated that quercetin (2) bound to the α-glucosidase active site. The benzene ring interacted with ALA329 and SER304 through π-alkyl and carbon–hydrogen bonds, respectively. The pyrone ring in the chromone structure interacted with VAL308. Additionally, the hydroxyl groups in the chromone structure formed conventional hydrogen bonds with THR310, ASP307, and ASP325.

The molecular docking model of emodin (**3**) is shown in Figure 5. It indicates that the trihydroxyanthraquinone ring formed a π-π T-shaped interaction with ILE328, ALA329, and VAL308 within the active site pocket. Additionally, the hydroxyl group interacted with ASP307 via a conventional hydrogen bond.

For further investigation of the binding mechanism between the active compounds and AChE active binding site, the potential compounds in Table 6, including emodin (**3**), chrysophanol (**4**), and physicon (**5**), were subjected to molecular docking study with the 3D structure of AChE (PDB code ID is 1C2B) from *Electrophorus electricus* [27]. Chlorogenic acid was used as a comparison.

**Figure 7:**
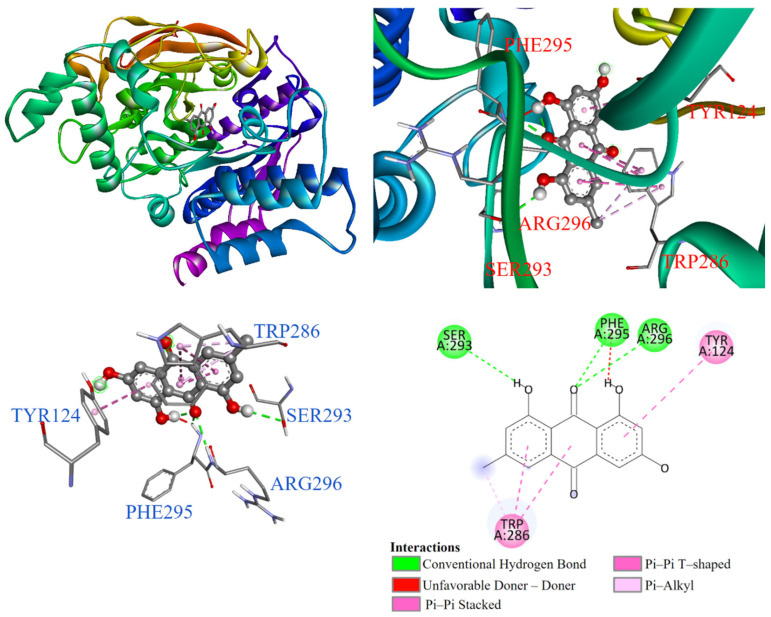
Interactions of emodin (**3**) with AChE active binding site.

As demonstrated in Figure 7, the molecular docking results showed that emodin (**3**) entered the substrate binding pocket with its chromone structure. The trihydroxyanthraquinone ring interacted through π-π stacking and π-π T-shaped interactions with TYR124 and TRP286, respectively, as well as a π-alkyl interaction. The hydroxyl and methyl groups formed conventional hydrogen bonds and an unfavorable donor–donor interaction with SER293, PHE295, and ARG296.

**Figure 8:**
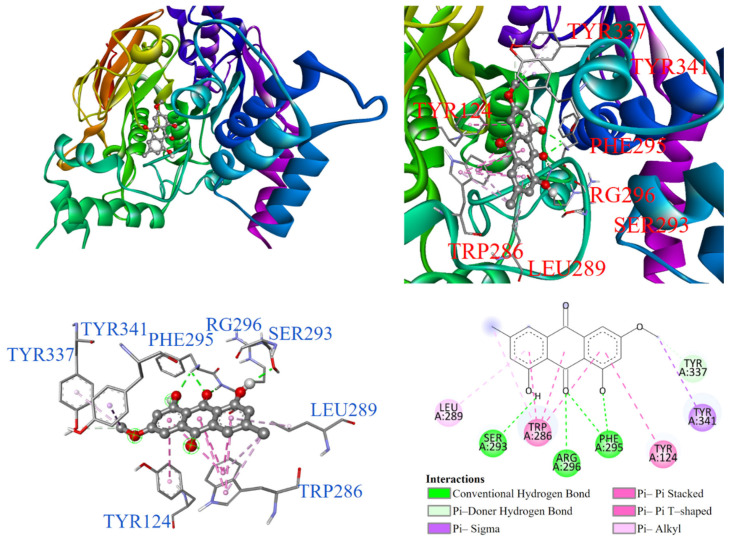
Interactions of physcion (**5**) with AChE active binding site.

As illustrated in Figure 8, the molecular docking results showed that physcion (**5**) enters the substrate binding pocket with its chromone structure. The dihydroxyanthraquinone ring interacted through π-π stacking, π-π T-shaped, and π-alkyl interactions with TYR124, TRP286, and LEU289, respectively. A methoxy group interacted via π-sigma and π-donor hydrogen bonding with TYR341 and TYR337. Additionally, the hydroxyl and methyl groups formed conventional hydrogen bonds with ARG296 and PHE295.

**Figure 9:**
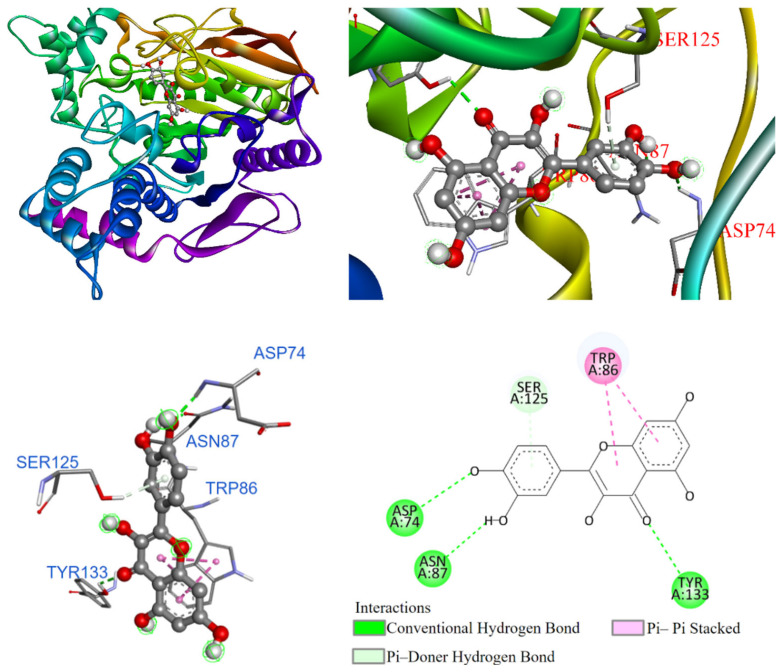
Interactions of quercetin (**2**) with AChE active binding site.

As illustrated in Figure 9, the molecular docking results showed that quercetin (**2**) entered the substrate binding pocket with its chromone structure. The trihydroxyanthraquinone ring interacts with TRP286 via π-π stacking. Additionally, the hydroxyl groups formed conventional hydrogen bonds with SER293, ARG296, and PHE295.

**Figure 10:**
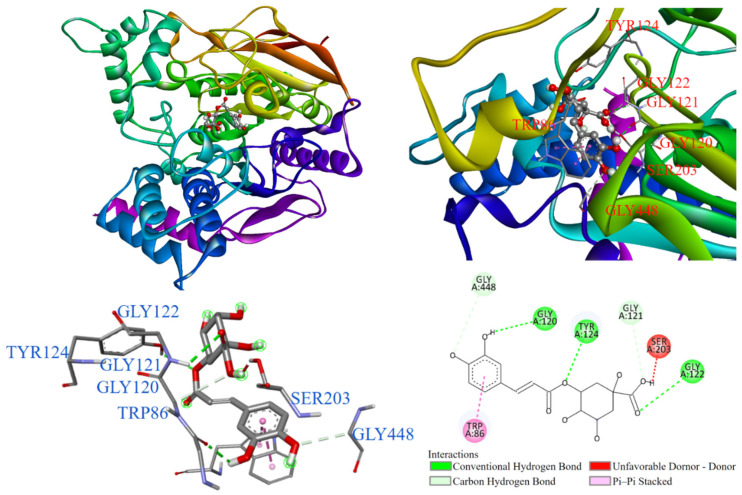
Interaction of chlorogenic acid with AChE active binding site.

As demonstrated in Figure 10, the molecular docking of the positive control showed that chlorogenic acid entered the substrate binding pocket with its benzene ring. The benzene ring interacted with TRP86 via π-π stacking, while the hydroxyl groups formed conventional hydrogen bonds, carbon–hydrogen bonds, and unfavorable donor–donor interactions with TYR124, GLY120, GLY122, GLY121, GLY448, and SER203. The presence of these unfavorable interactions in docking results may indicate repulsive forces between the ligand and target and destabilize the ligand–target complex in docking studies.

As shown in Table 9, the molecular docking results also indicated that physcion (**5**) (−9.7 kcal/mol) displayed significantly higher docking score than kaempferol (**1**) (−8.5 kcal/mol) and chlorogenic acid (−7.3 kcal/mol), indicating that physcion (**5**) possessed stronger binding affinity for AChE than other active components and positive control, consistent with the inhibition results in Table 6. These findings suggest that physcion (**5**) may be promising candidates for further investigation as a natural anti-AChE agent.

To study the mechanism of tyrosine inhibition, the most potent compounds in Table 7, including kaempferol (**1**) and quercetin (**2**), were evaluated through molecular docking to assess their binding ability to the crystal structure of tyrosinase. The 3D structure of tyrosinase (PDB ID: 2Y9X) was used as a docking model [6].

**Figure 11:**
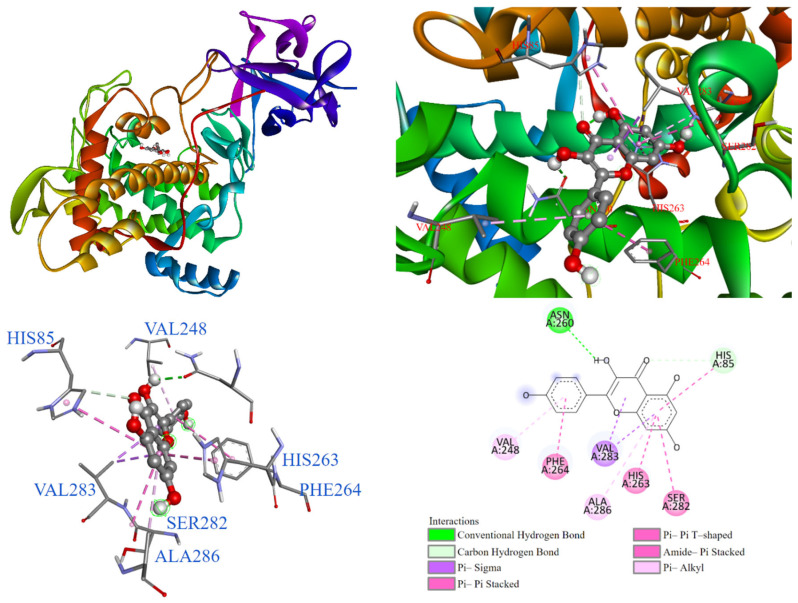
Interaction of kaempferol (**1**) with tyrosinase active binding site.

As demonstrated in Figure 11, the molecular docking model showed that kaempferol (**1**) entered the substrate binding pocket with its chromone structure. The benzene ring of the chromone structure interacted with copper ions, ALA286, SER282, HIS85, HIS263, and VAL283 through various interactions, including π-alkyl, π-π T-shaped, π-sigma, and amide-π stacking, respectively. The hydroxyl group on the benzene ring formed a carbon-hydrogen bond with HIS85 and a conventional hydrogen bond with ASN260. Additionally, the phenyl ring of the chromone structure interacted with PHE264 and VAL248 through π-alkyl, π-π T-shaped, and amide-π stacking interactions.

**Figure 12:**
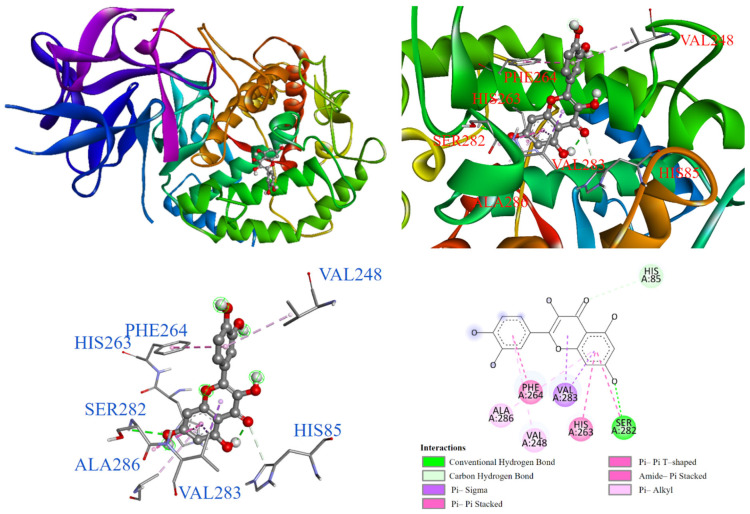
Interaction of quercetin (**2**) with tyrosinase active binding site.

In Figure 12, the molecular docking shows that quercetin (**2**) entered the substrate binding pocket with its chromone structure. The benzene ring of the chromone structure interacts with copper ions, HIS264, ALA286, VAL283, SER282, and HIS263 through various interactions, including π-alkyl, π-π T-shaped, π-sigma, and amide-π stacking, respectively. The hydroxyl group on the benzene ring formed a carbon–hydrogen bond with HIS85 and a conventional hydrogen bond with SER282. Additionally, the phenyl ring of the chromone structure interacted with PHE264 and VAL248 through π-alkyl, π-π T-shaped, and amide-π stacking interactions.

**Figure 13:**
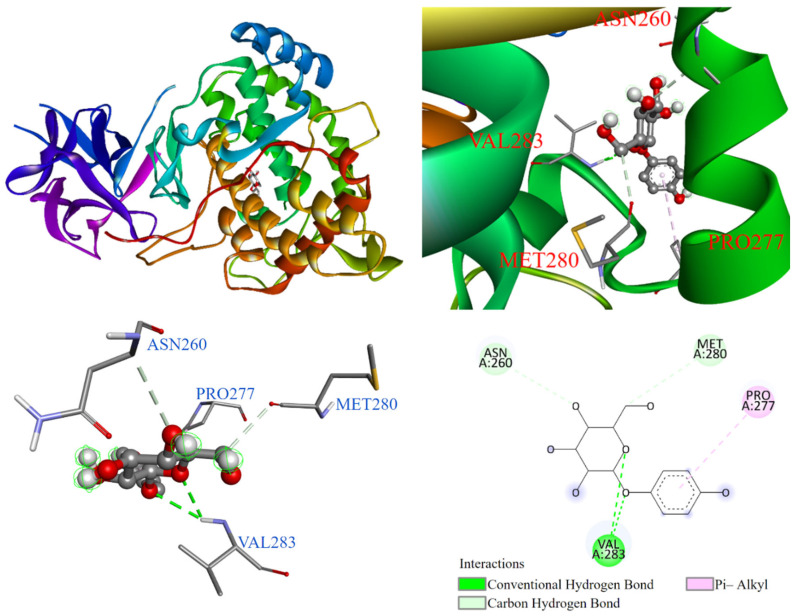
Interaction of arbutin with tyrosinase active binding site.

As shown in Figure 13, the positive control arbutin entered the substrate binding pocket with its benzene ring, which interacted with PRO277 via a π-alkyl interaction. Some of the hydroxyl groups on the glycosyl ring of arbutin formed interactions with MET280 and ASN260. Additionally, VAL283 formed a conventional hydrogen bond with the oxygen on the hydroquinone ring and the second oxygen on the six-carbon ring.

According to the molecular docking results in Table 10, the docking scores of kaempferol (**1**) (−7.8 kcal/mol) and quercetin (**2**) (−8.0 kcal/mol) were significantly higher than that of arbutin (−6.4 kcal/mol), indicating that kaempferol (**1**) and quercetin (**2**) can effectively bind to the tyrosinase binding site, consistent with the inhibition data in Table 7. These findings suggest that kaempferol (**1**) and quercetin (**2**) are worth further investigation as natural anti-tyrosinase agents.

## 4. Discussion

Considering challenges such as adverse effects and drug resistance, herbal medicines offer a promising alternative treatment [28]. With rich biological resources, economic feasibility, and easy access to natural products, it is promising to investigate the medicinal potential of herbs, plants, and fungi as candidates or health foods in Taiwan. Extraction of medicinal plants often involves the selection of suitable solvents to isolate the active ingredients or secondary metabolites [29,30]. In addition, factors such as plant material characteristics, solvent selection, solvent pH, and temperature also influence the extraction efficiency of active compounds [29,30,31]. Therefore, we used solvents of different polarities for extraction and evaluated the activity of bioactive components of *R. formosana*.

In the antioxidant (including DPPH, ABTS, superoxide radical scavenging, and FRAP) analysis results, various extracts of *R. formosana* revealed differing levels of antioxidant activity, similarly to the trend in TPC and TFC. However, the methanol and water extracts exhibited lower antioxidant activities in scavenging DPPH and ABTS. It may be attributed to the differences in TPC, TFC, or other antioxidant components in each extract. Additionally, kaempferol (**1**) and quercetin (**2**) showed stronger antioxidant activity than the positive control, BHT, indicating they can be used in the dietary supplement as natural antioxidants.

α-Glucosidase is located in the brush border of the small intestine and considered to be a therapeutic target for regulating postprandial hyperglycemia [32,33]. Acarbose is a standard antidiabetic drug and serves as a fundamental positive control in anti-α-glucosidase assays [34]. It can competitively delay carbohydrate digestion and absorption, thereby inhibiting postprandial hyperglycemia [35]. Each solvent extract of *R*. *formosana* demonstrated stronger α-glucosidase inhibition than the positive control. Additionally, kaempferol (**1**) and quercetin (**2**) exhibited superior α-glucosidase inhibitory activity compared to acarbose. The binding interactions between these isolated compounds and α-glucosidase further revealed the mechanism of α-glucosidase inhibition.

Alzheimer’s disease (AD) is a neurodegenerative disease that is characterized by progressive cognitive and memory decline [36,37]. It is often described as a phenomenon of accelerated brain aging [38]. In clinical therapy, it primarily uses acetylcholinesterase inhibitors to enhance cholinergic neurotransmission [6,27,39]. In this study, the ethyl acetate (EtOAc), acetone, and ethanol extracts demonstrated promising AChE inhibition. Among the bioactive components, quercetin (**2**), emodin (**3**), and physcion (**5**) exhibited notable AChE inhibitory activity, warranting further research as potential candidates for AD treatment. Furthermore, molecular docking results indicated that physcion (**5**) exhibited the highest binding energy (−9.4 kcal/mol), better than chlorogenic acid (−9.2 kcal/mol), suggesting that physcion (**5**) can effectively bind to the AChE active binding site.

Tyrosinase is the rate-limiting enzyme of regulating melanin related to the pigmentation disorders [40]. Recently, tyrosinase inhibitors have gained significance due to their potential applications in anticancer and prevention of pigmentation disorders [41]. In the anti-tyrosinase analysis results, the acetone extract of *R*. *formosana* exhibited higher tyrosinase inhibitory activity than other extracts. Notably, bioactive components including kaempferol (**1**) and quercetin (**2**) revealed approximately 50 times more potent tyrosinase inhibition than arbutin. Further molecular docking analysis confirmed high affinity of both kaempferol (**1**) and quercetin (**2**) for tyrosinase, aligning with the tyrosinase inhibition results.

## 5. Conclusions

This study demonstrates that the bioactive extracts and components from *R*. *formosana* exhibit potent antioxidant, anti-α-glucosidase, anti-tyrosinase, and anti-acetylcholinesterase activities through *in vitro* assays and *in silico* molecular docking analyses. The findings indicate the importance of selecting suitable solvents for extracting bioactive compounds, with ethyl acetate proving optimal for TFC extraction. Notably, water and 100 °C water extracts showed significant potential as natural antioxidants for health promotion and food preservation. Additionally, isolated compounds such as kaempferol (**1**), quercetin (**2**), and physcion (**5**) exhibited promising activities as natural anti-α-glucosidase, anti-AChE, and anti-tyrosinase agents. These results highlight the potential of bioactive extracts and components from the stem of *R. formosana* as natural sources for dietary supplements and therapeutic agents to manage oxidative stress-related conditions, including hyperglycemia, neurodegenerative diseases, and pigmentation disorders.

## Figures and Tables

**Table 1:** Extraction rates of TPC, TFC, and each extraction solvent for *R. formosana*.

Extracting Solvents	Relative Polarity	TPC (mg/g) ^a^ (GAE)	TFC (mg/g) ^b^ (QE)	Yields (%) ^c^
*n*-Hexane	0.009	<1	20.67 ± 3.13 **	0.23
Ethyl acetate	0.269	58.64 ± 4.39 *	25.07 ± 0.99 ***	1.54
Acetone	0.288	64.42 ± 0.41 ***	17.60 ± 3.12 *	2.79
Ethanol	0.355	59.98 ± 3.87 *	14.71 ± 1.66 **	2.91
Methanol	0.654	58.09 ± 4.69 *	8.82 ± 3.21 *	7.51
Water	0.762	41.44 ± 5.23	12.09 ± 2.93 *	4.09
100 °C Water	1.000	53.27 ± 1.44 ***	12.87 ± 3.12 *	9.85

^a^ Total phenolic content is expressed in milligrams of gallic acid equivalents (GAEs) per gram of extract. ^b^ Total flavonoid content is expressed in milligrams of quercetin equivalents (QEs) per gram of extract. ^c^ Yield is calculated as % yield = (extract weight/initial weight of dry sample) × 100. Statistical significance compared to the control is denoted as follows: *, **, and ***, respectively, represent *p* < 0.05, *p* < 0.01, and *p* < 0.001.

**Table 2:** Determination of free radical scavenging activity and FRAP assays.

Extracting Solvents	SC_50_ (μg/mL) ^a^			TE (mM/g) ^b^
	DPPH	ABTS	Superoxide	FRAP
*n*-Hexane	>400	>400	>400	19.82 ± 4.91
Ethyl acetate	34.91 ± 6.43	13.94 ± 3.26	169.88 ± 21.02 **	1096.56 ± 100.09
Acetone	39.76 ± 1.58	14.51 ± 1.19 *	50.26 ± 6.85 **	1252.35 ± 121.86
Ethanol	46.62 ± 9.48	14.52 ± 0.83 *	75.98 ± 1.93 ***	1463.77 ± 25.30 ***
Methanol	68.97 ± 8.89 **	18.35 ± 0.67 **	89.68 ± 1.79 ***	1273.00 ± 26.26 ***
Water	26.03 ± 1.95	21.25 ± 2.6 1 **	28.92 ± 2.37 *	1229.73 ± 80.01 **
100 °C Water	21.34 ± 0.52	13.63 ± 2.31	41.84 ± 1.40 **	1307.21 ± 4.19 ***
BHT ^c^	156.04 ± 14.31	9.12 ± 1.85	–	3196.39 ± 115.26 ***
Cynaroside ^d^	–	–	12.03 ± 4.20	–

^a^ SC_50_ value is defined as the sample concentration required to achieve 50% free radical scavenging. ^b^ FRAP results are expressed as Trolox equivalents (TEs) in mM per gram of extract. ^c^ BHT was used as the positive control for DPPH, ABTS, and FRAP analyses. ^d^ Cynaroside was used as the positive control for superoxide scavenging assay. *, **, and *** represent *p* < 0.05, *p* < 0.01, and *p* < 0.001, respectively.

**Table 3:** Determination of α-glucosidase and acetylcholinesterase inhibition.

Compounds	IC_50_ (μg/mL) ^a^	
	*α*-Glucosidase	AChE
*n*-Hexane	36.35 ± 6.00 **	181.86 ± 13.60
Ethyl acetate	3.19 ± 0.40 **	90.36 ± 7.28 **
Acetone	4.13 ± 0.53 **	84.66 ± 4.18 **
Ethanol	3.88 ± 1.06 **	63.23 ± 3.97 **
Methanol	2.51 ± 0.81 **	179.01 ± 12.89
Water	8.04 ± 1.72 **	169.66 ± 8.85
100 °C Water	7.88 ± 0.38 **	190.17 ± 11.26
Acarbose ^b^	523.62 ± 76.25	–
Chlorogenic acid ^b^	–	175.89 ± 12.33

^a^ IC_50_ values represent the concentration required to achieve 50% inhibition and are presented as mean ± SD (n = 3). ^b^ Acarbose and chlorogenic acid were used as positive controls. ** indicated *p* < 0.01 and was compared with the control.

**Table 4:** Tyrosinase inhibitory effects of each solvent extract.

Extracting Solvents	Tyrosinase IC_50_ (μg/mL) ^a^
*n*-Hexane	>400
Ethyl acetate	381.65 ± 10.57 ***
Acetone	310.16 ± 10.79 **
Ethanol	426.93 ± 14.86 *
Methanol	718.73 ± 54.07 **
Water	>400
100 °C Water	>400
Arbutin ^b^	162.42 ± 2.77

^a^ The IC_50_ value represents the concentration required to achieve 50% inhibition. ^b^ Arbutin was used as the positive control. Statistical significance compared to the control group is denoted as follows: * *p* < 0.05, ** *p* < 0.01, and *** *p* < 0.001. Data are presented as mean ± SD (n = 3).

**Table 5:** Components isolated from *R. formosana* were determined by antioxidant activity and FRAP.

Compounds	SC_50_ (μM) ^a^			TE (mM/g) ^b^
	DPPH	ABTS	Superoxide	FRAP
Kaempferol (**1**)	61.74 ± 7.36 **	15.32 ± 0.94 *	271.18 ± 20.69 **	1046.16 ± 23.78 ***
Quercetin (**2**)	16.05 ± 2.62 **	5.70 ± 0.92 **	85.80 ± 4.76 ***	1026.73 ± 16.69 ***
Emodin (**3**)	>200	>200	231.61 ± 15.91 **	<1
Chrysophanol (**4**)	>200	>200	>200	<1
Physcion (**5**)	>200	>200	>200	<1
Cynaroside ^c^	–	–	19.99 ± 1.70	–
BHT ^d^	708.15 ± 10.82	37.50 ± 4.31	–	5712.61 ± 176.27 ***

^a^ The SC_50_ value represents the sample concentration required to achieve 50% free radical scavenging. Values are presented as mean ± SD (n = 3). ^b^ FRAP results are expressed as Trolox equivalents (TE) in mM per gram of extract. ^c^ Cynaroside was used as the positive control for superoxide scavenging assay. ^d^ BHT was used as the positive control for DPPH, ABTS, and FRAP analyses. Statistical significance compared to the control group is denoted as follows: * *p* < 0.05, ** *p* < 0.01, and *** *p* < 0.001.

**Table 6:** Inhibitory activities of α-glucosidase and AChE isolated compounds.

Compounds	IC_50_ (μM) ^a^	
	*α*-Glucosidase	AChE
Kaempferol (**1**)	45.54 ± 3.20 ***	86.86 ± 4.62 **
Quercetin (**2**)	78.03 ± 8.69 ***	78.11 ± 2.87 **
Emodin (**3**)	58.85 ± 3.39 ***	77.56 ± 6.28 **
Chrysophanol (**4**)	604.33 ± 44.82 *	>400
Physcion (**5**)	>800	75.97 ± 1.34 **
Acarbose ^b^	406.91 ± 14.48	–
Chlorogenic acid ^b^	–	302.83 ± 25.24

^a^ The IC_50_ value was defined as half-maximal inhibitory concentration and was expressed as mean ± SD (n = 3); ^b^ Acarbose and chlorogenic acid were used as positive controls; * *p* < 0.05, ** *p* < 0.01, and *** *p* < 0.001 compared with the control.

**Table 7:** Effect of isolated compounds on tyrosinase inhibition.

Compounds	Tyrosinase IC_50_ (μM) ^a^
Kaempferol (**1**)	19.45 ± 1.82 **
Quercetin (**2**)	12.71 ± 1.96 **
Emodin (**3**)	>800
Chrysophanol (**4**)	>800
Physcion (**5**)	>800
Arbutin ^b^	638.73 ± 54.96

^a^ The IC_50_ value represents the half-maximal inhibitory concentration and was expressed as mean ± SD (n = 3). ^b^ Arbutin was used as positive control. ** indicated *p* < 0.01 and was compared with the control.

**Table 8:** Binding energies of active components and acarbose in *Saccharomyces cerevisiae* α-glucosidase.

Compounds	Affinity (kcal/mol)
Kaempferol (**1**)	–8.6
Quercetin (**2**)	–6.9
Emodin (**3**)	–7.7
Chrysophanol (**4**)	–3.8
Acarbose ^a^	–4.8

^a^ Acarbose used as positive control.

**Table 9:** Binding energies of bioactive compounds and chlorogenic acid in *E. electric* AChE.

Compounds	Affinity (kcal/mol)
Kaempferol (**1**)	–8.5
Quercetin (**2**)	–8.6
Emodin (**3**)	–9.4
Physcion (**5**)	–9.4
Chlorogenic acid ^a^	–7.3

^a^ Chlorogenic acid used as positive control.

**Table 10:** Binding energies of bioactive compounds and arbutin in tyrosine binding site.

Compounds	Affinity (kcal/mol)
Kaempferol (**1**)	–7.8
Quercetin (**2**)	–8.0
Arbutin ^a^	–6.4

^a^ Arbutin used as positive control.

## Data Availability

Data is contained within the article and Supplementary Material.

## Supplementary Materials

The following supporting information can be downloaded at: https://www.mdpi.com/article/10.3390/antiox14010008/s1, Figure S1: The ^1^H-NMR spectrum of kaempferol (**1**). Figure S2: The ^1^H-NMR spectrum of quercetin (**2**). Figure S3: The ^1^H-NMR spectrum of emodin (**3**). Figure S4: The ^1^H-NMR spectrum of chrysophanol (**4**). Figure S5: The ^1^H-NMR spectrum of physcion (**5**).
