# Supplementary material for: Antioxidant, Anti-α-Glucosidase, Anti-Tyrosinase, and Anti-Acetylcholinesterase Components from Stem of Rhamnus formosana with Molecular Docking Study
Source: Antioxidants (Basel). 2024 Dec 24;14(1):8. doi: 10.3390/antiox14010008 (PMC11761247; doi:10.3390/antiox14010008)
Supplement: Supplementary file 1 [file antioxidants-14-00008-s001.zip › antioxidants-3320205-supplementary.pdf]

Supplementary data

Antioxidant, Anti-α-glucosidase, Anti-tyrosinase, and Anti-acetylcholinesterase Components from Stem of *Rhamnus formosana* with Molecular Docking Study

Chia-Hsuan Tsai [1], Ya-Lun Liou [2,†], Sin-Min Li [2,†], Hsiang-Ruei Liao [3,4,5,*], and Jih-Jung Chen [2,6,7,*]

1. Department of Plastic and Reconstructive Surgery, Keelung Chang Gung Memorial Hospital, Keelung 204201, Taiwan; and College of Medicine, Chang Gung University, Taoyuan 333323, Taiwan; chtsai0715@gmail.com
2. Department of Pharmacy, School of Pharmaceutical Sciences, National Yang Ming Chiao Tung University, Taipei 112304, Taiwan; muset311386@gmail.com (Y.-L.L.); samuel147samuel147@gmail.com (S.-M.L.)
3. Graduate Institute of Natural Products, College of Medicine, Chang Gung University, Taoyuan 333323, Taiwan; liaoch@mail.cgu.edu.tw
4. Graduate Institute of Biomedical Sciences, College of Medicine, Chang Gung University, Taoyuan 333323, Taiwan
5. Department of Anesthesiology, Chang Gung Memorial Hospital, Taoyuan 333323, Taiwan
6. Department of Medical Research, China Medical University Hospital, China Medical University, Taichung 404333, Taiwan
7. Traditional Herbal Medicine Research Center, Taipei Medical University Hospital, Taipei 110301, Taiwan

---

* Correspondence: jjungchen@nycu.edu.tw (J.-J.C.), liaoch@mail.cgu.edu.tw (H.-R.L.); Tel.: +886-2-2826-7195 (J.-J.C.); Fax: +886-2-2823-2940 (J.-J.C.)

† These authors contributed equally to this work.

Contents

Supplementary Fig. 1. The ^{1}H-NMR spectrum (400 MHz, methanol-d_4) of kaempferol (**1**)……. S3

Supplementary Fig. 2. The ^{1}H-NMR spectrum (400 MHz, acetone-d_6) of quercetin (**2**)………… S3

Supplementary Fig. 3. The ^{1}H-NMR spectrum (400 MHz, acetone-d_6) of emodin (**3**) ………….. S4

Supplementary Fig. 4. The ^{1}H-NMR spectrum (400 MHz, chloroform-*d*) of chrysophanol (**4**)... S4

Supplementary Fig. 5. The ^{1}H-NMR spectrum (400 MHz, chloroform-*d*) of physcion (**5**)……... S5

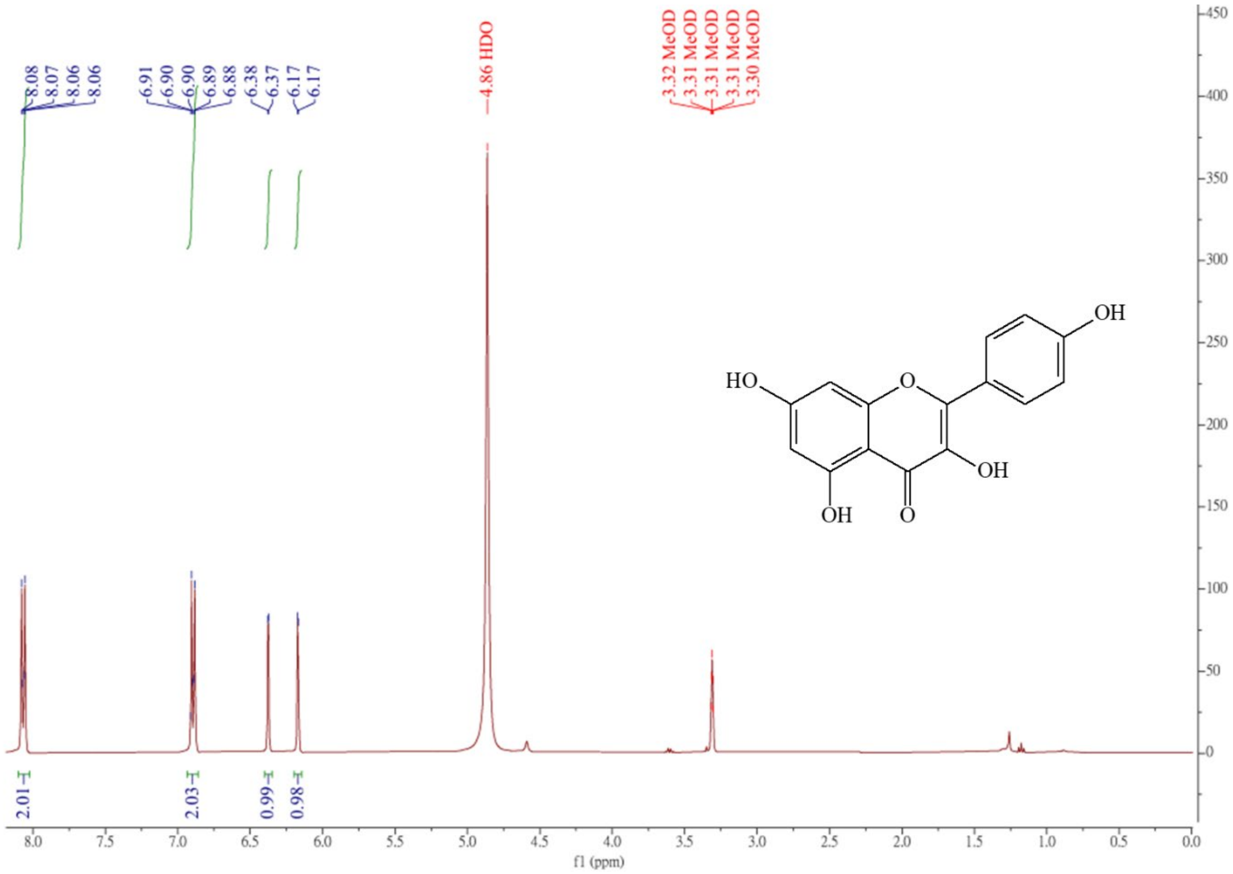

Supplementary Fig. 1. The ^{1}H-NMR spectrum (400 MHz, methanol-d_4) of kaempferol (**1**).

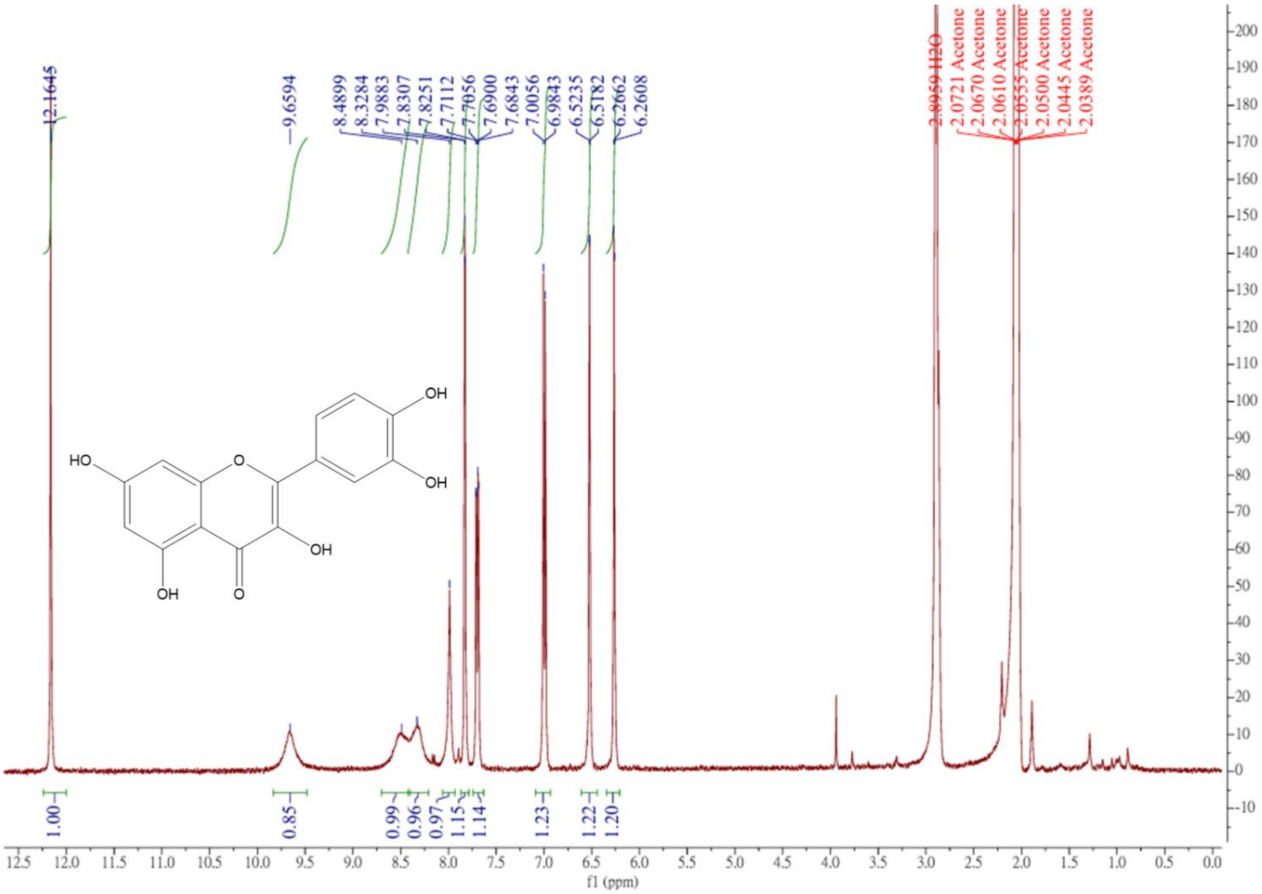

Supplementary Fig. 2. The ^{1}H-NMR spectrum (400 MHz, acetone-d_6) of quercetin (**2**).

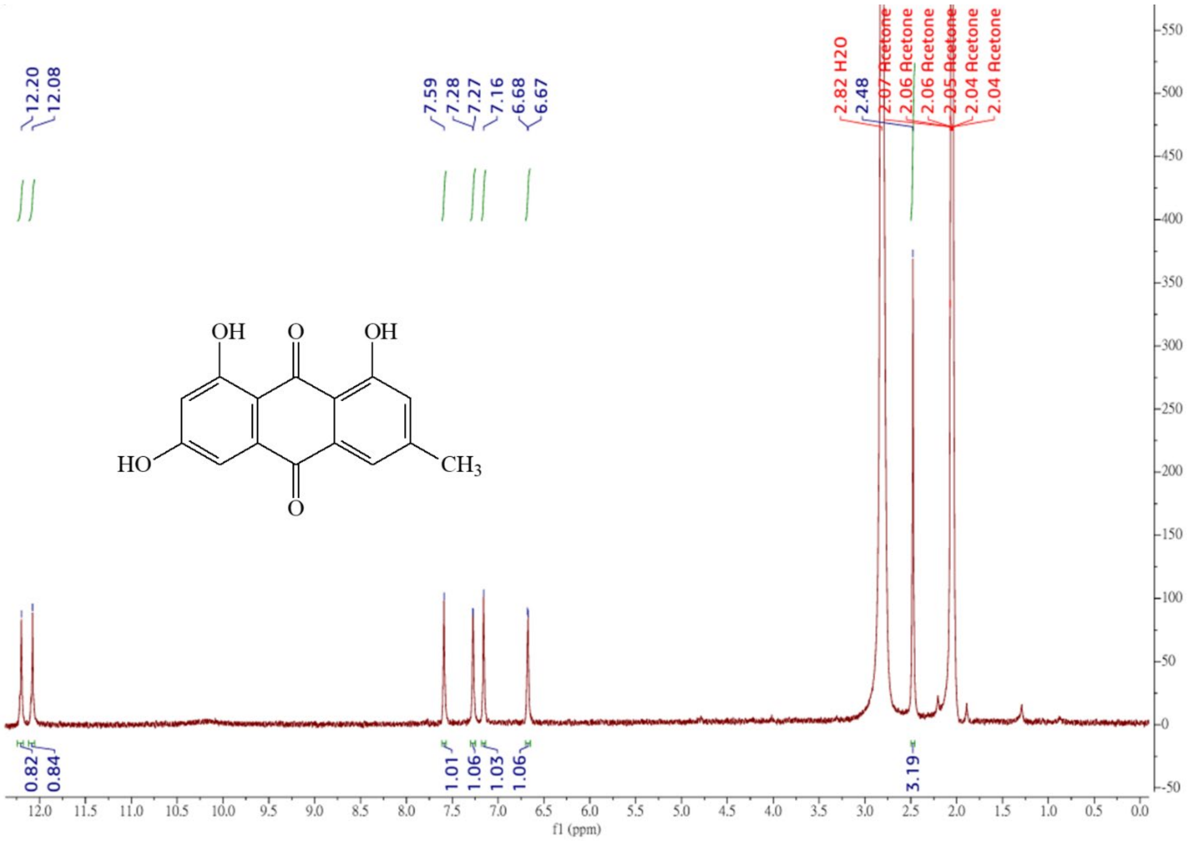

Supplementary Fig. 3. The ^{1}H-NMR spectrum (400 MHz, acetone-*d*6) of emodin (**3**).

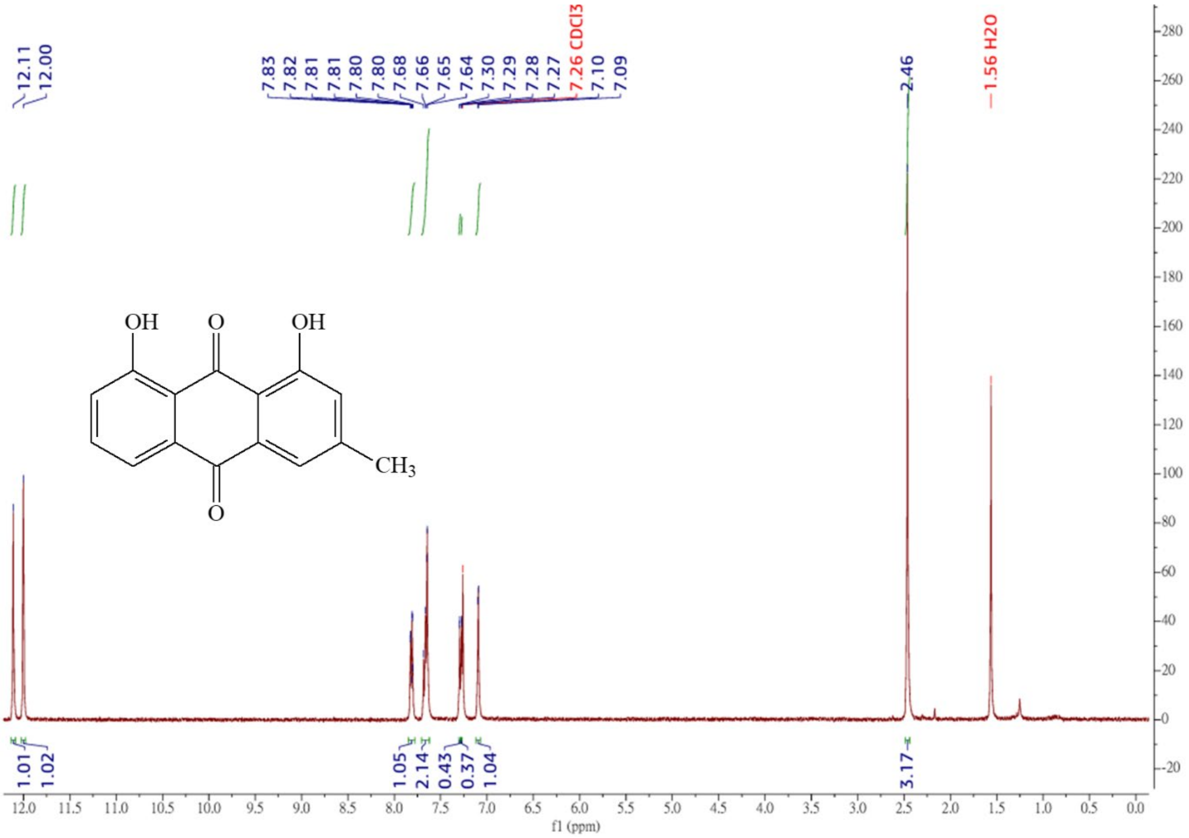

Supplementary Fig. 4. The ^{1}H-NMR spectrum (400 MHz, chloroform-*d*) of chrysophanol (**4**).

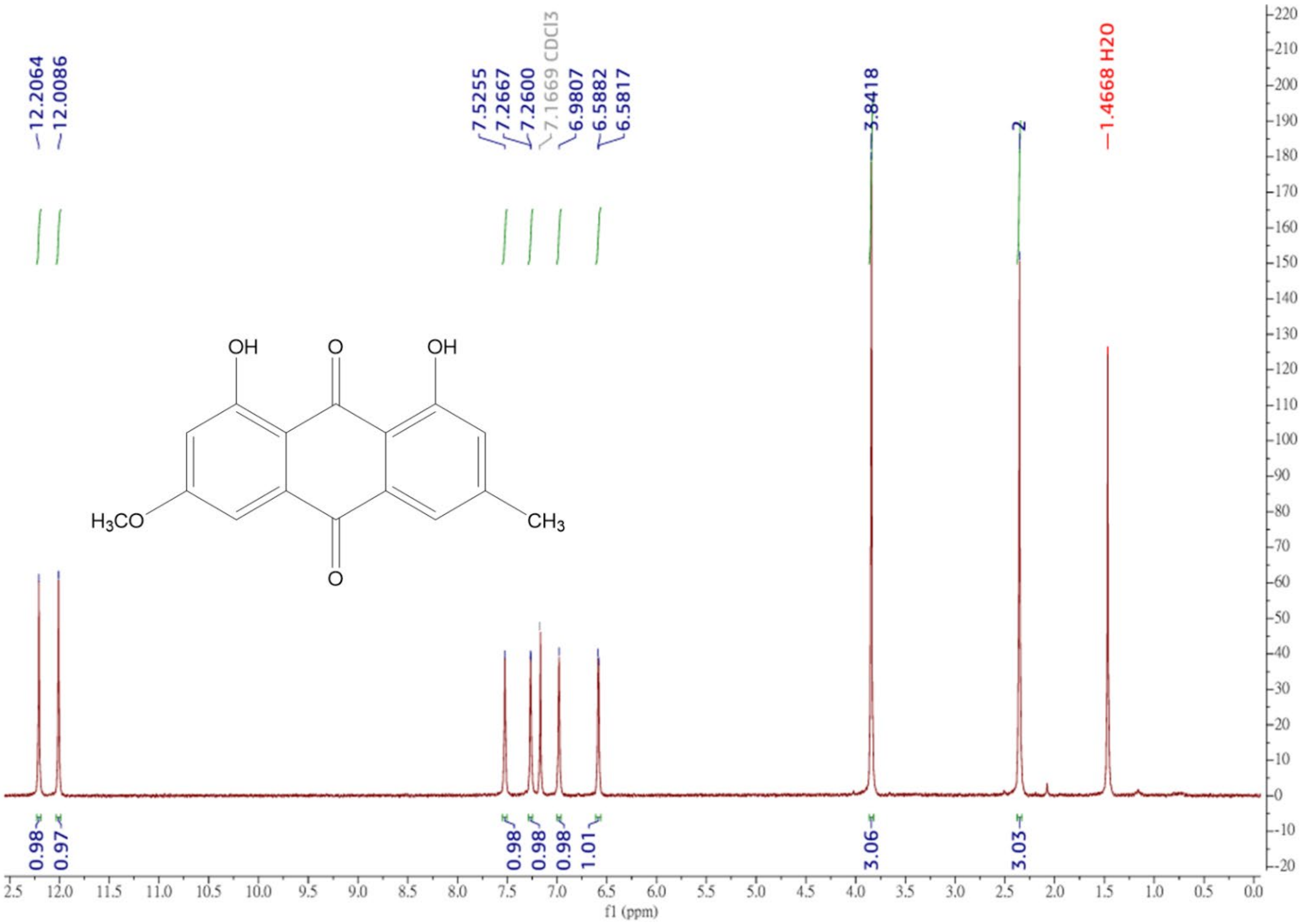

Supplementary Fig. 5. The ¹H-NMR spectrum (400 MHz, chloroform-*d*) of physcion (**5**).
